# Ambiguity and Conflict Aversion When Uncertainty Is in the Outcomes

**DOI:** 10.3389/fpsyg.2019.00539

**Published:** 2019-03-29

**Authors:** Michael Smithson, Daniel Priest, Yiyun Shou, Ben R. Newell

**Affiliations:** ^1^ Research School of Psychology, The Australian National University, Canberra, ACT, Australia; ^2^ School of Psychology, University of New South Wales, Sydney, NSW, Australia

**Keywords:** uncertainty, ambiguity aversion, conflict aversion, risk, decision

## Abstract

We argue that the way ambiguity has been operationalized throughout the literature on ambiguity effects has an important limitation, insofar as ambiguity in outcomes has been neglected. We report two studies where judges do encounter ambiguity in the sampled outcomes and find evidence that ambiguity aversion is not less than when judges are given a range of outcomes without reference to ambiguous outcomes themselves. This result holds regardless of whether people are presented with a sample all at once or sample outcomes sequentially. Our experiments also investigate the effects of conflicting information about outcomes, finding that conflict aversion also does not decrease. Moreover, ambiguity and conflict aversion do not seem to arise as a consequence of judges ignoring uncertain outcomes and thereby treating outcome sets as reduced samples of unambiguous (or unconflicting) information. Instead, ambiguity and conflict aversion are partly explained by more pessimistic outcome forecasts by judges. This pessimism, in turn, may be due to the judges’ uncertainty about how the chance of a desirable outcome from an ambiguous or conflictive alternative compares with an equivalent risky alternative. Both studies used hypothetical scenarios, and no incentives were provided for participants’ decisions.

The typical study of ambiguity effects in the judgment and decision-making literature operationalizes ambiguity in the form of a summary description using a range, e.g., of the number of balls in an urn that has the same color. This type of description was initially set in Ellsberg’s (1961) pioneering paper. Recent extensions of studies of ambiguity by several researchers to include “experienced” ambiguity have incorporated operationalizations of ambiguity that permits sequential sampling of outcomes, in place of a summary description. Ert and Trautmann (2014) use a fixed but unknown probability of a “favorable” outcome. Dutt et al. (2014) have underlying uniform distributions on the integers from 0 to 100 and from 1 to 100 that determine the probability of a favorable outcome. Güney and Newell (2015) employ three second-order distributions for determining the probability of a favorable outcome on each turn. These probabilities and distributions are unknown to the participants, but they can learn them if they draw sufficiently large samples. As Dutt et al. (2014, p. 318, 325) point out, their distinction between risk and ambiguity is “one random variable in decisions under risk and two or more nested random variables in decisions under ambiguity” and they observe that this differs from the classic Ellsberg distinction.

Both the traditional and these new operationalizations of ambiguity are limited to unambiguous *outcomes*. Smithson (2015) argues that in the three experimental setups described above, judges do not actually experience ambiguity in the samples that they draw because every sampled case is unambiguous (e.g., each ball from an urn has a specific color). Thus, the judge simply learns a probability distribution by sampling unambiguous stimuli, and the sole manifestation of “ambiguity” is in the second-order distribution or additional nested random variable, which is unknown to (and not experienced by) the judge but eventually learned by the judge as if it is just another first-order distribution. For all of these setups, sampling reduces ambiguity.

Smithson (2015) proposes that ambiguous outcomes ought to be a focus of investigations into ambiguity effects. In this approach, judges would be unsure about the status of some outcomes as they encounter them. For example, a judge in a medical testing scenario may receive inconclusive (ambiguous) test results or diagnoses. This operationalization relates to traditional conceptualizations of ambiguity (Knight, 1921; Ellsberg, 1961) but locates it in the outcomes themselves. It is not clear whether people are affected by ambiguous outcomes in the same way as they are by probability distributions, but we hypothesize that ambiguity aversion will not disappear when people are presented with ambiguous outcomes.

Following suggestions by Smithson (2015), we extend the outcome-based operationalization of uncertainty to include conflicting information about outcomes. “Conflicting information” here refers to inputs from multiple sources that disagree with one another (e.g., one doctor says a test indicates the presence of a pathogen, whereas another doctor says the test does not). Conflicting information need not be ambiguous, and of course, ambiguous information need not be conflicting. For instance, two forecasters could agree that the probability of rain tomorrow is between 0.3 and 0.5 (ambiguous but non-conflicting information); and another two may disagree, the first stating a probability of 0.3 and the second a probability of 0.5 (unambiguous but conflicting information). Notably, both conflict and ambiguity can represent equivalent levels of uncertainty, as in these examples.

Several studies of uncertainty arising from summarized conflict have found that there is a greater aversion to conflicting information than to ambiguous information (e.g., Smithson, 1999; Cabantous, 2007; Cabantous et al., 2011). A natural question is whether manifestations of conflict aversion hold to the same degree when conflicting information is summarized in a description of a sample of outcomes (e.g., one doctor says 18 of 20 blood tests indicate pathogens, whereas another says only 12 of 20 indicate pathogens) versus when it is elaborated in each outcome (e.g., when we can see whether the two doctors agree or disagree on each of the tests).

Conflict aversion has been manifested in two ways. First, a majority of people prefer to receive or deal with messages from ambiguous rather than conflicting sources (Smithson, 1999, 2013). Second, people tend to make more pessimistic best estimates for future outcomes under conflict than ambiguity (Viscusi, 1997; Cabantous, 2007; Cabantous et al., 2011). This may be due to people feeling that they must choose one or another of the conflicting estimates and that the most conservative estimate is a prudent choice. Viscusi and Chesson (1999) present evidence for this hypothesis. A similar effect has been hypothesized and/or reported in the literature on ambiguity (e.g., Hurwicz, 1951; Einhorn and Hogarth, 1985), and indeed Ert and Trautmann note that “ambiguous prospects are typically associated with pessimistic weighting” (Ert and Trautmann, 2014, p. 40). Given the evidence that conflict is dispreferred to ambiguity, it is possible that this may be partly due to people making more pessimistic estimates under conflict than they do under ambiguity, so a subsidiary aim in our experiments is to compare people’s predictions under these two types of uncertainty.

Finally, we need to clarify the concept of “outcome elaboration.” There are two common kinds of realistic sampling situations. In some settings, samples are presented all at once (e.g., a set of examination scores from a class, or a batch from an assembly line for quality-control testing). In others, people build up samples sequentially, outcome by outcome. The latter kind of sampling may be susceptible to sequence effects, such as working-memory limits and recency effects, although Hau et al. (2010) compared participant preferences and responses in one-by-one versus all-at-once samples and found little or no difference between them. Accordingly, in our studies, we investigate both kinds of sampling setups, but we begin with all-at-once samples to ensure that memory limitations will not have an effect on decisions.

The guiding hypothesis for the studies reported here is that both ambiguity and conflict aversion will occur when these uncertainties are located in outcomes sampled from a population, regardless of whether the outcomes are summarized in a description or displayed individually, because sampling does not reduce *outcome* ambiguity or conflict. As reported in the literature (e.g., Shou and Smithson, 2015), we expect that samples incorporating only risk (i.e., where all outcomes have clear statuses or properties) will be preferred to ambiguity and conflict (i.e., where information about the statuses of some outcomes is either ambiguous or conflicting) and that ambiguity will be preferred to conflict. However, an open question is whether the strength of ambiguity or conflict aversion will be the same under summarization versus elaboration *via* a sample.

Two studies are reported here. Study 1 compares ambiguity and conflict attitudes when such information is summarized versus when it is elaborated (in all-at-once samples), and Study 2 compares these attitudes when sampling is sequential versus when the sample is presented all at once.

## Study 1

Study 1 employs a hypothetical scenario, which presents participants with two decisions, one involving a choice between alternative pairs of art authentication experts (the sources of information) and the other being a choice between two art bequests that have been assessed by the experts:

You are the head curator of an art gallery which has recently received two collections of modern artworks at the bequest of two patrons. Each collection contains 20 artworks. You want to hang each collection without separating the works, however currently you only have the space to hang one collection. The two collections are of an equal importance, both boasting the work of several prominent 20^th^ century artists. You have decided to hang whichever collection has the greater percentage of genuine artworks from prominent artists compared to artworks which are forgeries or incorrectly attributed to a prominent artist. You have hired four authentication experts to work in pairs on each collection to analyse the works and determine if they are genuine.

Participants were asked which bequest they would choose to hang in the gallery, and which pair of experts they would prefer to hire to authenticate any future bequests. They also were asked to estimate the number of genuine artworks in each bequest.

As suggested earlier, the major hypothesis regarding subjective outcome probabilities or forecast estimates is that people will produce more pessimistic or conservative estimates under conflict or ambiguity than under risk. A related hypothesis is that more pessimistic estimates will predict stronger aversion, and a relevant open question is whether differences in estimates under different kinds of uncertainty can entirely account for the preference for one uncertainty over another. An exploratory question raised earlier is whether estimates under conflict are more pessimistic than under ambiguity. If they are, then that could be a candidate explanation for the preference of ambiguity over conflict.

A final consideration is whether people will simply ignore ambiguous or conflictive data in the samples and base their judgments and decisions solely on the unambiguous data. If they do, then the resulting smaller samples under ambiguity and conflict might account for a preference for risk. This does not seem plausible, given the consistent findings that people prefer ambiguity to conflict, and a direct test of this under ambiguity by Shou and Smithson (2015) showed that people did not ignore ambiguous information when making causal inferences. Nonetheless, we include the following diagnostic test here.

Smithson (2015) observes that there is more than one defensible “best” estimate of a probability when the evidence to hand contains favorable, unfavorable, and indeterminate outcomes. Let *f*_1_ be the number of favorable outcomes out of *N* samples and *f*_2_–*f*_1_ the number of indeterminate outcomes. The “midpoint” best estimate of the probability of a favorable outcome, *p*_1_, is $p_{1}=\left(f_{1}+f_{2}\right)/2N\text{.}$ However, a reasoner could base a best estimate solely on the unambiguous data, producing $p_{1}=\left(f_{1}/N\right)/\left(1-f_{2}/N+f_{1}/N\right)\text{.}$ As Smithson (2015) points out, while we would expect people to use the midpoint estimate when presented with a probability interval (i.e., when uncertainty is summarized), it is plausible that they might use the unambiguous data (UD) estimate when presented with a sample (i.e., when uncertainty is located in the outcomes). For instance, if there are *f*_1_ = 6 favorable outcomes out of *N* = 20, and *f*_2_–*f*_1_ = 4 uncertain outcomes, then *f*_2_ = 10, and so the midpoint estimate would be $p_{1}=\left(f_{1}+f_{2}\right)/2N=16/40=0.4$, whereas the UD estimate would be $p_{1}=\left(f_{1}/N\right)/\left(1-f_{2}/N+f_{1}/N\right)=6/16=0.375\text{.}$ The UD estimate is lower than the midpoint when the midpoint is less than 0.5 and higher when the midpoint exceeds 0.5. We therefore included conditions in Study 1 with the midpoint below 0.5, at 0.5, and above 0.5. If people are ignoring ambiguous and/or conflictive data, then this will be apparent in their subjective probability estimates.

A summary of the hypotheses and exploratory questions for Study 1 is as follows:

H1. Ambiguity aversion and conflict aversion will be observed regardless of whether uncertain information about the artworks is summarized or outcome elaborated.
H2. Ambiguity (conflict) aversion will increase as the amount of ambiguity (conflict) in the information increases.
H3. Best estimates will be more pessimistic under ambiguity and conflict than under risk.

H1 restates our major claim, namely that sampling is insufficient to reduce ambiguity or conflict aversion when the outcomes are the locus of these uncertainties. H2 and H3 are ancillary hypotheses derived earlier from the literature. Additional exploratory questions to be investigated are as follows:

Q1. Does degree of ambiguity or conflict aversion depend on whether uncertain information is summarized or outcome elaborated?
Q2. Are best estimates more pessimistic under conflict than under ambiguity?

### Methods

#### Participants

Participants were 470 North American adults recruited through Qualtrics. There were 241 females and 229 males. Ages ranged from 18 to 87 years with a mean of 48.45 (*SD* = 14.34). All participants were paid $7.50 USD by Qualtrics. Participant decisions in experimental tasks did not incur any financial rewards or penalties. The data showed no evidence of bots or fraudulent entries.

#### Design

To test the relationship between source preferences, best estimates, and decisions, a mixed-factorial design was employed: 2 (summarized versus outcome elaborated) × 2 (40% of cases uncertain versus 20% of cases uncertain) × 3 (the midpoint number of genuine artworks being 30 versus 50 versus 70% of all artworks) × 3 (risk-ambiguity comparison versus risk-conflict comparison versus ambiguity-conflict comparison). The midpoint number factor was between-subjects, and all other factors were within-subjects. Participants were randomly assigned to one of the three midpoint number conditions and completed one task in each of the remaining conditions in random order, with each participant completing 2 × 2 × 3 = 12 tasks.

#### Procedure

For each condition, the qualities of the information given by the authentication experts for each bequest of 20 artworks were manipulated. Two kinds of uncertainty were given in each trial. Ambiguity aversion was studied in the risk-ambiguity comparison and conflict aversion in the risk-conflict comparison, where one source conveyed risk information (“The pair of experts authenticating Bequest X came to consensus on all of the artworks”), while the other source either conveyed ambiguous information (“The pair of experts authenticating Bequest X were unsure about the authorship of some of the artworks”) or conflicting information (“The pair of experts authenticating Bequest X disagreed about the authorship of some of the artworks”), respectively. Conflict aversion relative to ambiguity was studied under ambiguity-conflict comparisons *via* the same stimuli.

Within each condition, participants were given a range of values for the true number of genuine artworks. This is referred to as the *uncertainty interval*. The midpoint and uncertainty level conditions map to the location and width of this interval and generate different frequentist predictions for the best estimates. For example, consider the 40% uncertainty condition with 20 artworks and a midpoint of 14 (a 70% midpoint). Because 40% of 20 is 8, that is the number of uncertain outcomes. Given the midpoint of 14, the uncertain interval therefore is [10, 18] genuine works.

In the *summarized-description* condition, participants were presented with a verbal and numerical outcome description, such as the following (50% midpoint and 20% uncertainty condition):

*Risky*: The pair of experts authenticating Bequest A came to consensus on all of the artworks. The experts judged that 10 of the 20 artworks are genuine.
*Ambiguous*: The pair of experts authenticating Bequest B was unsure about the authorship of some of the artworks. The experts judged that between 8 and 12 of the 20 artworks are genuine.
*Conflicting*: The pair of experts authenticating Bequest B disagreed about the authorship of some of the artworks. One expert judged that 8 of the 20 artworks are genuine, whereas the other expert judged that 12 of the 20 artworks are genuine.

In the *outcome-elaborated* condition, information about each artwork was given simultaneously in Table 1.

Table 1 displays the risky and ambiguous versions of the art bequest assessments. A1–A20 and B1–B20 refer to individual artworks assessed by each expert in the bequest presented on this trial. In the conflictive version, the “Undetermined” entries in the table were replaced with “Genuine” in one column and “Not genuine” in the other. For clarity, each table was color coded (represented in gray scale in Table 1) to provide further visual cues about the outcomes in each condition. The order of outcomes in each table was randomized and pre-generated; however, each table started with a positive outcome (“Genuine”) and ended with a negative outcome (“Not genuine”) to prevent recency- and primacy-effect confounds from differing initial and final outcomes between conditions.

**Table 1:** Outcome-elaborated condition.

	Expert A1	Expert A2		Expert B1	Expert B2
A1	Genuine	Genuine	B1	Genuine	Genuine
A2	Not genuine	Not genuine	B2	Not genuine	Not genuine
A3	Genuine	Genuine	B3	Undetermined	Undetermined
A4	Not genuine	Not genuine	B4	Not genuine	Not genuine
A5	Genuine	Genuine	B5	Genuine	Genuine
A6	Genuine	Genuine	B6	Genuine	Genuine
A7	Genuine	Genuine	B7	Genuine	Genuine
A8	Not genuine	Not genuine	B8	Not genuine	Not genuine
A9	Genuine	Genuine	B9	Genuine	Genuine
A10	Not genuine	Not genuine	B10	Not genuine	Not genuine
A11	Genuine	Genuine	B11	Undetermined	Undetermined
A12	Not genuine	Not genuine	B12	Undetermined	Undetermined
A13	Not genuine	Not genuine	B13	Not genuine	Not genuine
A14	Not genuine	Not genuine	B14	Not genuine	Not genuine
A15	Genuine	Genuine	B15	Genuine	Genuine
A16	Genuine	Genuine	B16	Genuine	Genuine
A17	Not genuine	Not genuine	B17	Not genuine	Not genuine
A18	Genuine	Genuine	B18	Genuine	Genuine
A19	Not genuine	Not genuine	B19	Undetermined	Undetermined
A20	Not genuine	Not genuine	B20	Not genuine	Not genuine

Left: the assessments of two experts given Bequest A in the risky-outcome version of the art curator scenario; right: the opinions of two different experts given Bequest B in the ambiguous-outcome version.

Forced-choice preferences for information sources (“Which pair of experts would you prefer to hire to authenticate any future bequests?”) and for outcome sets (“Which bequest would you choose to hang in the gallery?”) were used to determine attitudes toward ambiguity and conflict. For both information source preferences and outcome-set preferences, written free-response questions requested participants to justify or explain their decisions (not analyzed or reported here). Best estimates of the number of genuine artworks were elicited using sliders with a range of 0–20 for both bequests immediately prior to choosing the preferred outcome set but after choosing the preferred information source.

### Results

Source preferences (which pair of experts participants would hire) and bequest preferences (which set they would hang in the gallery) were very strongly associated. Overall, 91.7% of bequest and source preferences were the same. This did not differ when the information was summarized (91.5%) versus outcome elaborated (91.9%); nor did it vary substantially across uncertainty comparison types (93.8% for risk versus ambiguity, 93.1% for risk versus conflict, and 88.4% for ambiguity versus conflict). Because the experimental variables had such similar effects on source and bequest preferences in logistic regression models, we present only the source preference models here.

To test our hypotheses, we estimated random-intercept logistic regression models, with the form $y_{ij}^{\prime }=\beta_{0j}+\cdots +\varepsilon_{ij}\text{,}$ where $y\prime_{ij}$ is the logit of the predicted probability, $\beta_{0j}=\gamma_{0}+u_{j}$ with $u_{j}∼N\left(0,\sigma_{u}^{2}\right)\text{,}$ and $\varepsilon_{ij}∼N\left(0,\sigma_{e}^{2}\right)\text{.}$ Experimental variables were treated as fixed-effects variables. Because each participant completed 2 × 2 × 3 = 12 tasks, each main-effect term had either 4 or 6 data points per condition and interaction terms either 2 or 3 data points per cell for estimating subject-specific effects. These are under-sized groups, and no random-effects models converged other than random-intercept-only models.

#### Source Preferences

The test of hypothesis H1 (that ambiguity and conflict aversion will be present regardless of whether the uncertain information is summarized or outcome elaborated) revealed that ambiguity and conflict aversion were present in both the summarized and outcome-elaborated conditions. Thus, hypothesis H1 was supported. The best logistic regression model fixed-effects part is

(1) $$\begin{array}{l} y\prime_{ij}=\gamma_{0}+\beta_{12}a_{i}+\beta_{13}c_{i}+\beta_{2}d_{i}+\beta_{122}a_{i}d_{i}+\beta_{132}c_{i}d_{i\;}\\ \;=-2.328+0.171a_{i}+1.410c_{i}-0.151d_{i}+0.102a_{i}d_{i}+0.641c_{i}d_{i} \end{array}$$

where *a* and *c* are binary {0, 1} variables identifying the ambiguity versus risk and conflict versus ambiguity pairs, respectively, and *d* = 1 for the outcome-elaborated condition and 0 for the summarized-description condition. The dependent variable is the probability of hiring the experts from Bequest B, which is the ambiguous case for the ambiguity-risk pair, and the conflictive case for the other two pairs involving conflicting experts.

Q1 (whether ambiguity and conflict aversion differ between the summarized versus outcome-elaborated conditions) also was investigated in this model, *via* the interaction terms. The interaction terms significantly improved model fit over the corresponding main-effects-only model (*χ* ^2^(2) = 15.022, *p* < 0.001). The model showed no significant difference between the summarized and outcome-elaborated conditions for the risk versus ambiguity or risk versus conflict choices (*z* = −1.026, *p* = 0.305). However, the preference for ambiguity over conflict was somewhat stronger in the summarized than in the outcome-elaborated condition (*z* = 3.435, *p* = 0.001). These results can be interpreted *via* the odds in the right-hand column of Table 2.

**Table 2:** Frequencies, probabilities, and odds of choosing Source A over Source B.

	Source A	Source B	
Comparison type	*n* (prob)	*n* (prob)	Odds
**Summarized**			
A = risk versus B = ambiguity	785 (0.84)	153 (0.16)	5.131
A = risk versus B = conflict	768 (0.82)	170 (0.18)	4.518
A = ambiguity versus B = conflict	610 (0.65)	328 (0.35)	1.860
**Outcome elaborated**			
A = risk versus B = ambiguity	799 (0.85)	139 (0.15)	5.748
A = risk versus B = conflict	773 (0.82)	165 (0.18)	4.685
A = ambiguity versus B = conflict	535 (0.57)	403 (0.43)	1.328

Hypothesis H2 [ambiguity (conflict) aversion will increase with the amount of ambiguity (conflict) in the stimuli] was tested by adding the uncertainty variable to the model in equation (1) and testing for both main and interaction effects. The best model is as follows:

(2) $$\begin{array}{l} y\prime_{ij}=\gamma_{0}+\beta_{12}a_{i}+\beta_{13}c_{i}+\beta_{2}d_{i}+\beta_{3}u_{i}+\beta_{122}a_{i}d_{i}\\ \;+\beta_{132}c_{i}d_{i}+\beta_{123}a_{i}u_{i}+\beta_{133}c_{i}u_{i}\\ \;=-2.135+0.028a_{i}+1.147c_{i}-0.152d_{i}-0.411u_{i}\\ \;+0.104a_{i}d_{i}+0.644c_{i}d\;+0.303a_{i}u_{i}+0.549c_{i}u_{i} \end{array}$$

where *u_i_* is the two-level uncertainty factor. This model significantly improves model fit over the model in equation (1) (*χ* ^2^(3) = 9.835, *p* = 0.020). The preference for risk over ambiguity is higher when uncertainty is greater (odds = 6.401 when uncertainty = 40% and 4.697 when uncertainty = 20%), but this effect does not occur for preferences of risk over conflict (odds = 4.802 when uncertainty = 40% and 4.434 when uncertainty = 20%) or ambiguity over conflict (odds = 1.500 when uncertainty = 40% and 1.648 when uncertainty = 20%). H2 therefore is supported only for the risk versus ambiguity comparison.

#### Best Estimates

We investigated the effects of uncertainty type and summarized versus elaborated outcomes on participants’ estimates of the true number of genuine artworks. To begin, we found that very few participants appeared to use the unambiguous-data estimates (4.4% of the responses for the conditions with the midpoint at 14 of 20). Thus, participants were not ignoring the ambiguous or conflictive cases in the samples. Instead, participants used either the midpoint of the uncertainty interval, the interval’s lower bound, a value midway between the midpoint and the lower bound, or some other value.

**Figure 1:**
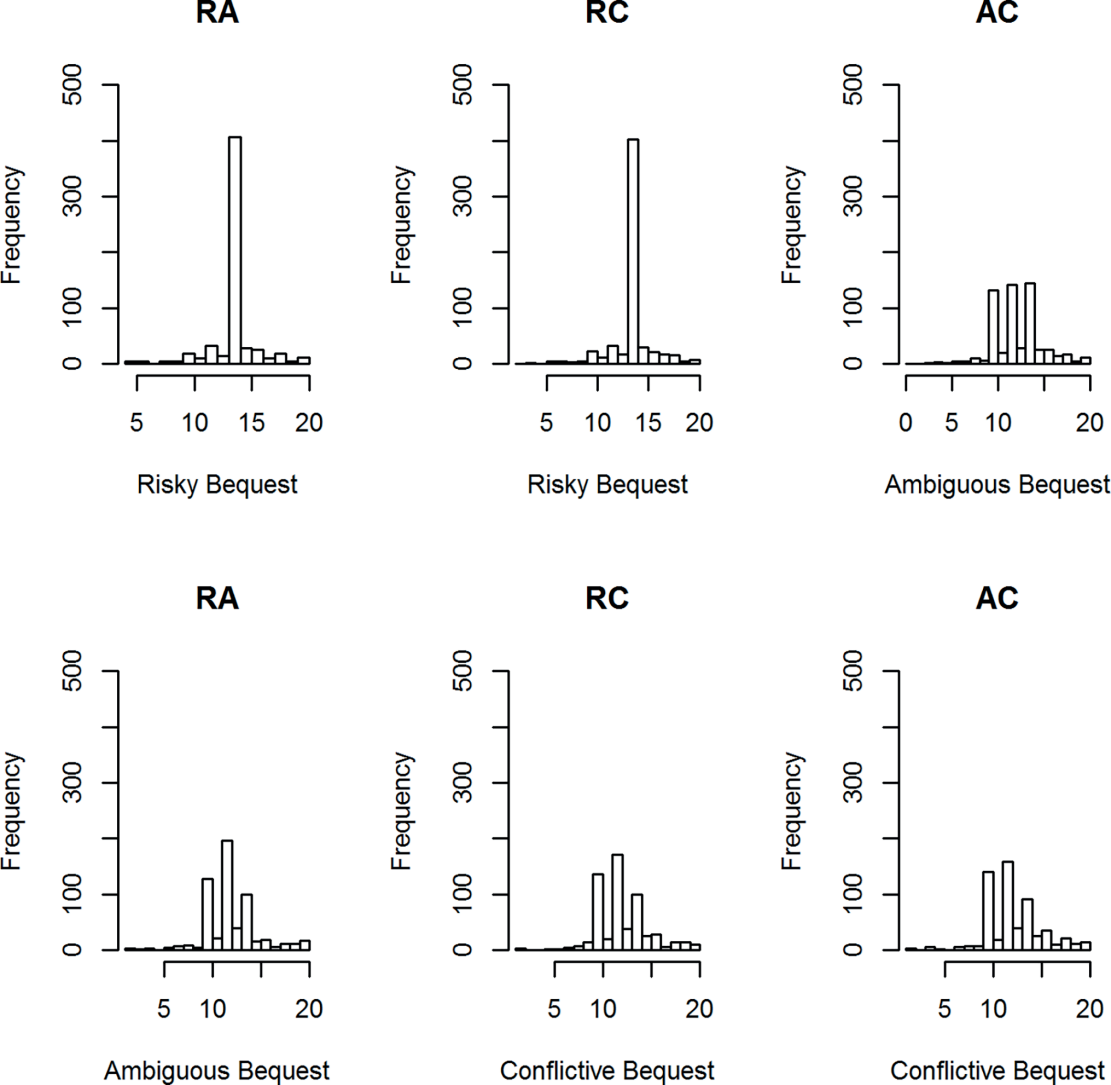
Histograms of best estimates of the number of genuine artworks for three conditions: RA = risk versus ambiguity, RC = risk versus conflict; AC = ambiguity versus conflict.

Figure 1 displays a representative condition, the histograms of participants’ best estimates when the midpoint number of genuine artworks was 14 and the uncertainty interval was [10, 16]. There are four clear component distributions in the data: peaks at 10, 12, and 14, and a widely dispersed distribution of other values. The “midpoint” peak at 14 is prominent in the Risky Bequest graphs. The peaks at 10 and 12 are indicative of pessimistic estimates of the number of genuine works, given the uncertainty interval [10, 16]. These appear along with a peak at 14 in the Ambiguous and Conflictive Bequest graphs.

Our approach was to ascertain the joint effect of the type of uncertainty (risk, ambiguity, or conflict), and whether the uncertainty is summarized or outcome elaborated on which of four alternatives participants chose for their “best estimate” of the number of genuine artworks in the bequest. While the technically “best” analysis would be a four-component mixture model with three degenerate component distributions and a beta distribution to capture the remaining component (e.g., Smithson et al., 2011), we opted for binary logistic regressions that better suited our purposes by testing three contrasts:

1. midpoint versus lower bound and midway point
2. midway point versus lower bound
3. other value versus everything else

The first two contrasts were designed to compare how conservative or pessimistic participants were about the number of genuine artworks under different experimental conditions, and the third was intended to detect differences in the frequency of non-modal choices across experimental conditions. We briefly describe the main results here; details of the analyses and results are available in the Supplementary Materials.

In line with hypothesis H3 (more pessimistic estimates under ambiguity and conflict than under risk), conflict and ambiguity evoked more pessimistic estimates than risk did, and this effect was amplified under outcome elaboration. These were large effects and presented the most marked distinctions between summarization and outcome elaboration in this study. However, there was no clear evidence for more pessimistic estimates under conflict than under ambiguity, thereby yielding only partial support for H3. Table 3 displays the frequencies with which best estimates fell into the four categories (lower bound, midway point, midpoint, and other), and the odds corresponding to the three contrasts.

**Table 3:** Frequencies and odds of the four best estimate choices.

	Other	Low B.	Midw. P.	Midpt.	Odds_1_	Odds_2_	Odds_3_
**Summarized**							
Risk	407	70	52	1,351	11.074	0.743	0.276
Ambiguity	592	562	160	556	0.760	0.285	0.460
Conflict	647	645	159	429	0.534	0.247	0.525
**Outcome elaboration**							
Risk	556	48	63	1,213	10.928	1.313	0.420
Ambiguity	536	922	182	240	0.217	0.197	0.399
Conflict	659	826	162	233	0.236	0.196	0.540

Note: Odds_1_ = Midpt./(Low B. + Midw. P.); Odds_2_ = Low B./Midw. P.; Odds_3_ = Other/(Low B. + Midw. P. + Midpt.).

We begin with the midpoint versus lower-bound or midway-point contrast. The mixed logistic regression testing this contrast in the summarized-versus-elaborated and the uncertainty conditions yielded main effects for ambiguity and conflict versus risk and interaction terms for ambiguity and conflict by summarized-versus-elaborated.

These effects may be interpreted *via* the odds_1_ column in Table 3. The odds of choosing the midpoint versus midway or lower bound as best estimate under risk are 11.074 for the summarized condition and 10.928 for the outcome-elaborated condition, whereas the odds of doing so under ambiguity or conflict all are far below 1. The odds ratios in the odds_1_ column for comparing risk with the other two uncertainties are markedly larger in the outcome elaborated than in the summarized condition. The risk-ambiguity odds ratio is 11.074/0.760 = 14.571 for the summarized condition, whereas it is 10.928/0.217 = 50.359 for the elaborated condition; and the risk-conflict ratio is 11.074/0.534 = 20.738 for the summarized condition, whereas it is 10.928/0.236 = 46.305 for the elaborated condition.

This interaction effect arises from the fact that the difference in the popularity of the midpoint between the risk and the other two uncertainty types is much greater in the outcome-elaborated than in the summarized-description condition. The other effects regarding Odds_2_ and Odds_3_ in Table 3 are of subsidiary importance here and are detailed in the Supplementary Materials.

#### Effect of Best Estimates on Preferences

Given the effects of uncertainty type and summarized-versus-elaborated status on estimates of the number of genuine artworks, a natural question to raise is whether the estimates themselves might account for the choices between experts or bequests. Accordingly, we investigated whether the sign and magnitude of the difference, best estimate A – best estimate B, would predict participants’ choices. We also were interested in whether any evidence of ambiguity or conflict aversion, or differences between summarized and outcome-elaborated conditions, would remain when these estimate-differences were taken into account.

If the risky alternative estimate is larger than the sign is positive, whereas if the ambiguous or conflictive estimate is larger than the sign is negative. We evaluated random-intercept logistic regression models that incorporated binary dummy variables representing positive and negative differences (there were cases where differences were 0, so both dummy variables were required), products of the two dummy variables, and the absolute magnitude of the difference. Table 4 displays the model coefficients, standard errors, and significance tests.

**Table 4:** Mixed logistic regression fixed effects for the choice of experts to hire.

Predictor	Coeff.	Std. err.	*z*	*p*
Intercept	−2.199	0.144	−15.324	<0.001
Risk-conflict	0.067	0.110	0.608	0.543
Ambiguity-conflict	1.205	0.108	11.196	<0.001
Outcome elaborated	0.292	0.082	3.569	<0.001
Positive difference	−0.888	0.168	−5.296	<0.001
Negative difference	0.890	0.178	5.011	<0.001
Positive difference × |difference|	−0.063	0.040	−1.566	0.117
Negative difference × |difference|	0.177	0.039	4.573	<0.001

Note: The positive difference and negative difference variables take values {0, 1}, and |difference| is the absolute value of the difference.

The negative intercept gives the log-odds of preferring the ambiguous to the risky source (i.e., the odds of choosing the ambiguous over the risky source are exp(−2.199) = 0.111). The risk-versus-conflict coefficient is small (0.067), so, as before, the odds for this comparison are very similar to those for risk-versus-ambiguity. Likewise, the ambiguity-conflict odds remain much as in our earlier model, but its moderation by summarized-versus-elaborated status has disappeared when differences between estimates are taken into account. On the other hand, a main effect for summarized-versus-elaborated status has emerged, such that both ambiguity and conflict aversion are moderately diminished in the outcome-elaborated condition but certainly do not vanish (the odds ratio is exp(0.292) = 1.339, so the odds of choosing the ambiguous or conflicting source increase, e.g., for ambiguity from 0.111 to 0.148).

The effects of estimate differences are in the expected direction, so that a positive difference increases conflict and ambiguity aversion, while a negative difference decreases these aversion effects. The impact of difference magnitude is substantially greater for negative than for positive differences, as can be seen in the positive difference × |difference| and negative difference × |difference| coefficients in Table 4.

### Study 1 Discussion

Study 1 demonstrates that ambiguity aversion and conflict aversion do not vanish when ambiguous or conflicting information is outcome-elaborated rather than summarized, at least when a sample of information is presented all at once. The results also suggest that estimates of the extent of favorable outcomes (in this case, number of genuine artworks in a bequest) are more pessimistic under ambiguity and conflict than under risk, with this effect being more strongly manifested when information is outcome-elaborated than when it is summarized. There was little evidence of such a difference between estimates for the conflictive and ambiguous sources.

## Study 2

The goals of Study 2 were fivefold, although we focus on just two goals here. The first goal was to investigate whether ambiguity and/or conflict aversion occur when people experience ambiguity or conflict *via* sequential sampling, and whether the strength of aversion differs from when the entire sample is available versus as it is accumulated. Two versions of sequential sampling therefore were tested, one requiring participants to remember earlier samples and the other not requiring the use of memory. The purpose of this manipulation was to examine whether the effects of ambiguity or conflict diminish under the memory-requirement condition, possibly because of inaccurate recall or recency effects. A recent study of causal reasoning under ambiguity found evidence that greater cognitive load decreased (but did not eliminate) the effect of ambiguity (Shou and Smithson, 2015). However, based on the findings in Hau et al. (2010) and our findings in Study 1, it is also plausible that neither requiring memory nor sequential sampling itself would have an effect on ambiguity or conflict aversion.

The second goal, motivated by findings in Study 1, was to examine the impact of sampling format on subjective outcome estimates, and the extent to which these estimates would predict preferences. From the findings in previous relevant studies (e.g., Viscusi, 1997; Cabantous, 2007) and in Study 1, our primary hypotheses were that ambiguity and conflict would result in more pessimistic estimates, which in turn would predict preferences in the same way as was found in Study 1. That is, more pessimistic (or conservative) outcome estimates will yield greater ambiguity or conflict aversion. However, in Study 2, we tested this latter hypothesis by ascertaining how well prior estimates predict post-outcome preferences and judgments.

There were three additional goals in Study 2 and corresponding hypotheses, but these are not directly relevant to this paper and therefore are elucidated in the Supplementary Materials. The art bequest scenario was not well-suited to sequential sampling or to a “revealed truth” (i.e., whether an artwork is provably genuine or not) without relatively complicated scenarios. We elected to employ a weather-forecast scenario instead. Weather is naturally sequentially sampled, and, of course, any weather forecast eventually can be compared against how the weather turned out. Weather forecasting also readily lends itself to instances of ambiguity and/or disagreements among alternative forecasts. This study therefore utilized weather-forecast scenarios as the vehicle for testing the hypotheses. The scenarios and their characteristics are described in Methods section.

In summary, the major hypotheses for Study 2 are as follows:

H1. Ambiguity and conflict aversion will be present in all types of sampling.
H2. Ambiguous and conflicting forecasts will result in more pessimistic (or conservative) outcome estimates than risky (i.e., unambiguous and unconflicting) forecasts.
H3. More pessimistic (conservative) prior estimates will increase post-outcome ambiguity and conflict aversion.

### Methods

#### Participants and Design

The sample consisted of 567 North American adult participants recruited through Qualtrics (279 females and 288 males). All were paid $6.75 USD by Qualtrics. Participant decisions in experimental tasks did not incur any financial rewards or penalties. Eleven participants were excluded due to completing the study in an unreasonably short amount of time (<6 min; *MN* = 16.0, *SD* = 9.9). The data showed no evidence of bots or fraudulent entries. The mean age was 44.03 (*SD* = 15.39). The majority of participants spoke English as their first language (508 of 556).

The experiment used a hypothetical weather forecast scenario in which participants were required to evaluate weather forecasts from two independent forecasting companies. Participants were instructed as follows:

- Your business relies on weather forecasts to decide if an event should be canceled due to rain. You have been examining two alternative companies that offer weather-forecasting services and are responsible for providing advice about which company to use. Your boss asks you to collect 12 forecasts from each of the forecasters.
- Because you can only afford to pay for one forecast on each day, you have to collect them one by one, by clicking the button to purchase each forecast. Each click will reveal one forecast result.

There were four types of forecast: predicting a rainy day, predicting a sunny day, ambiguous, and conflicting. Ambiguous forecasts stated that “no clear forecast can be provided,” while conflicting forecasts stated that “the company’s models provided disagreeing forecasts.” Appropriate sun and rain icons were displayed accompanying the forecast statements.

The study had a 3 (types of comparison) × 2 (the number of ambiguous [conflicting] cases) × 2 (probabilities of the focal event) × 2 (types of sampling) × 2 (order of presentation) mixed-factorial design. The between-subject factors were the type of comparison (risk versus conflict, ambiguity versus conflict, and ambiguity versus conflict), the types of sampling (memory dependent versus memoryless), and the order of presentation of the two companies. The within-subject factors were the probability of rain (1/2 versus 2/3), and the amount of uncertainty (low versus high number of ambiguous or conflicting forecasts). These factors are explained below. Participants were randomly assigned to 1 of the 12 conditions (the sample size per cell ranged from 36 to 57).

#### Materials and Procedures

There were three types of company. The *risky* company provided only rainy or sunny forecasts. The *ambiguous* company provided rainy, sunny, or agreed ambiguous forecasts. The *conflictive* company provided rainy, sunny, or conflicting forecasts. Three types of comparison were ambiguity-risk, conflict-risk, and ambiguity-conflict. The ambiguity-risk pair was used to test ambiguity aversion. The conflict-risk and ambiguity-conflict pairs were used to test conflict aversion.

Each comparison condition included four scenarios, classified by the probability of the focal event in the forecasts [P(Rain): 1/2 versus 2/3] and the number of ambiguous/conflicting cases (Alevel: low versus high). The distribution of stimuli in each scenario is shown in Table 5.

**Table 5:** Stimuli distribution across conditions.

Forecasts (outcomes)*						
Scenario	Alternative	Sunny	Rainy	Amb./Confl.	P(Rain)	Alevel
1	Risk	6 (3S + 3R)	6 (3S + 3R)	0 (N/A)	1/2	High
	Amb/Confl	1 (0S + 1R)	1 (1S + 0R)	10 (6S + 4R)		
2	Risk	6 (3S + 3R)	6 (3S + 3R)	0 (N/A)	1/2	Low
	Amb/Confl	4 (2S + 2R)	4 (2S + 2R)	4 (2S + 2R)		
3	Risk	4 (2S + 2R)	8 (4S + 4R)	0 (N/A)	2/3	High
	Amb/Confl	1 (0S + 1R)	2 (0S + 2R)	9 (6S + 3R)		
4	Risk	4 (3S + 1R)	8 (4S + 4R)	0 (N/A)	2/3	Low
	Amb/Confl	3 (2S ± 1R)	6 (3S ± 3R)	3 (1S ± 2R)		

* The first number is the number of forecasts, and the numbers in brackets are the outcomes. For instance, in Scenario 1, the Risk company forecasted 6 sunny days of 12 days, whereas the actual outcomes on those 6 days were 3 sunny and 3 rainy (3S + 3R) days.

In the first part of the task, participants observed forecast results from the paired two companies *via* self-paced sampling. In each within-subject condition, two tables containing forecast results were presented in vertical order on a single page. Each table accommodated one company’s forecast results and was accompanied by a sampling button. The order of the two alternatives on the page was randomized across different participants (i.e., served as the between-subject order of presentation factor). Participants clicked the sampling button and revealed one forecast at a time. They could freely alternate between the two companies in the sampling sequence (i.e., which company’s forecast would be revealed in each trial). In each block, after exhaustively sampling all forecast results from the paired alternatives, participants were required to estimate the number of sunny days according to each alternative forecaster and asked to indicate which forecaster they preferred. As in Study 1, there were no material consequences for their estimates or decisions, i.e., participant payments did not depend on their decisions.

There were two types of sampling: memoryless and memory-dependent. In the memoryless condition, the revealed forecast results accumulated and stayed in the forecast result table. Participants were able to observe all forecast results after finishing sampling. The forecasts in the memory-dependent condition were shown only once when sampled and did not stay on screen when the next forecast was revealed.

**Figure 2:**
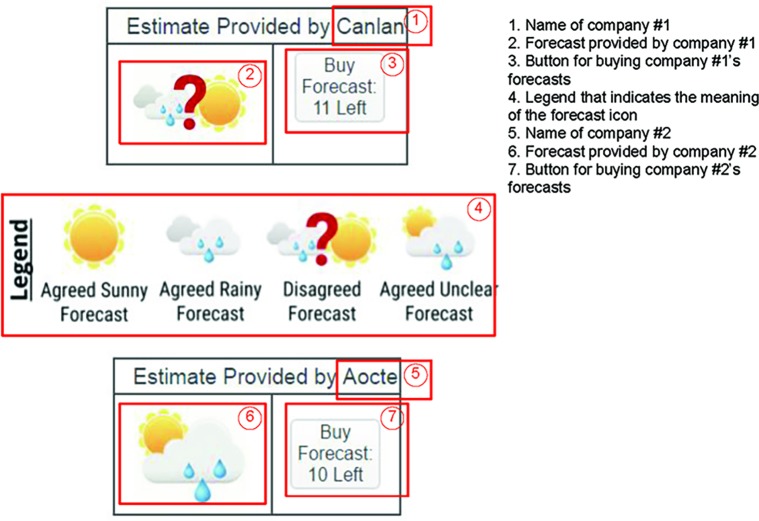
Example stimuli for the memory-dependent condition.

Figure 2 displays example stimuli for the memory-dependent condition. Participants observed two companies’ forecasts as shown in the figure: One for company No. 1 and one for company No. 2. In this illustration, the participant clicked the button in ③ to purchase a forecast provided by the company No. 1, which is shown in cell ②. Each time the participant pressed ③, a new forecast icon would display in ②. The button in ③ also displays the number of forecasts remaining to be sampled. The initial number of forecasts is 12 and reduces by 1 each time the participant presses ③ When all forecasts from company No. 1 have been purchased, the button in ③ will be disabled and will display the text “all purchased.” Similarly, each time the participant clicks ⑦, a new forecast provided by the company No. 2 would display in ⑥ The number in ⑦ starts from 12 and reduces by 1 each time the participant presses ⑦ until all forecasts have been purchased.

**Figure 3:**
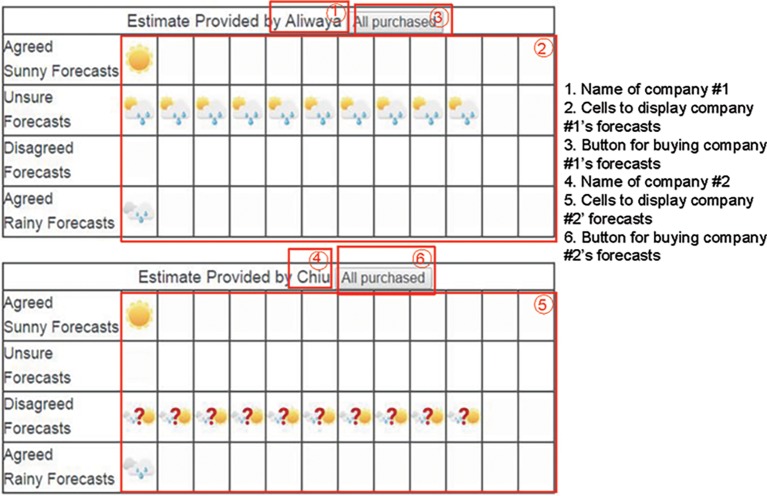
Example stimuli for the memoryless condition.

Figure 3 displays example stimuli for the memoryless condition. Similar to the memory-dependent condition, participants click the button in ③ to purchase a forecast provided by the company No. 1 and click ⑥ to purchase a forecast provided by the company No. 2. Forecasts from company 1 are displayed in cells in ② according to the type of forecasts, while forecasts from company 2 are displayed in cells in ⑤. The two tables start with empty cells and add a forecast icon each time participants click the purchase button. The icon stays in the table and additional icons appear when more forecasts are purchased. The figure shows the screen when all forecasts have been purchased, and the participant is able to observe all forecasts in one single screen.

**Figure 4:**
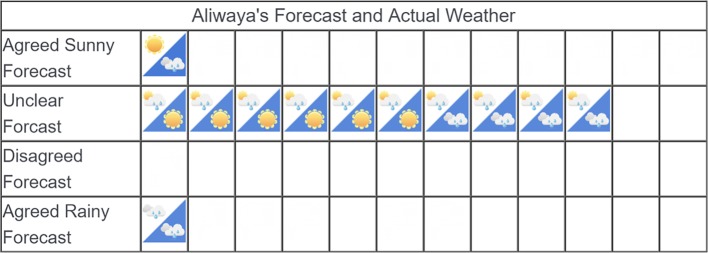
Example post-outcome stimuli.

In the second part of the task, participants in both sampling conditions were shown the actual weather outcomes in comparison to the company’s forecasts. Figure 4 shows sample post-outcome stimuli for Company No. 1 in Figure 3. In each cell that displays an icon, the upper left triangle shows the forecast of the company (the same as those in Figure 2), and the lower right triangle with blue background shows the weather outcome.

The frequencies of the outcomes are shown in the bracketed figures in Table 5. Participants were then asked which company they preferred and also to nominate which forecast they thought was the more accurate. Although the probability of rain in the forecasts was set at 1/2 and 2/3, the actual outcomes were evenly split between rainy and sunny days. The purpose of maintaining an even split between the outcomes was to explore whether the worse accuracy for the 2/3 condition would have an impact on ambiguity or conflict aversion (we had no hypothesis about this effect).

### Results

As in Study 1, ambiguity and conflict aversion were found in Study 2. Table 6 displays the logistic regression estimated odds of preferring risk over ambiguity, risk over conflict, and ambiguity over conflict, in choices of forecasters both prior-to- and post-outcomes, and likewise for judgments of which forecast was the more accurate. As in Study 1, these are random-intercepts models, with the form $y\prime_{ij}=\beta_{0j}+\varepsilon_{ij}\text{,}$ where *yʹ_ij_* is the logit of the predicted probability, $\beta_{0j}=\gamma_{0}+u_{j}$ with $u_{j}∼N\left(0,\sigma_{u}^{2}\right)\text{,}$ and $\varepsilon_{ij}∼N\left(0,\sigma_{e}^{2}\right)\text{.}$ The odds then are $\exp\left(\gamma_{0}\right)\text{.}$ All of the odds in Table 6 are substantially (and significantly) greater than 1, so the first hypothesis is supported.

**Table 6:** Prior-to- and post-outcome forecast and accuracy preference odds.

	Prior	Post-outcome	
Forecast pair	Forecast	Forecast	Accuracy
Risk versus ambiguity	3.631	2.927	2.581
Risk versus conflict	6.702	4.557	4.764
Ambiguity versus conflict	4.430	6.458	6.791

We will consider only prior-to-outcome forecast preferences and post-outcome accuracy choices for the remainder of the paper. This is because, as foreshadowed above, the forecasts that participants chose as most accurate also were the ones they preferred once the outcomes were known (the relevant model odds ratios for risk versus ambiguity, risk versus conflict, and ambiguity versus conflict were 591.068, 455.209, and 159.804, respectively). No other independent variables had any impact when accuracy choice was included in the model for post-outcome preferences.

We now investigate whether the type of sampling influenced the degree of ambiguity or conflict aversion. The models used to test this were main-effect random-intercepts models of the form $y\prime_{ij}=\beta_{0j}+\beta_{1}s_{i}+\varepsilon_{ij}\text{,}$ with *β*_0*j*_ as defined above and *s* denoting the sampling format. These models were compared against the null models described above, *via* likelihood-ratio tests. Prior-to-outcome forecast preferences were not significantly influenced by sampling format (the likelihood-ratio tests yielded $\chi_{1}^{2}=2.125$, *p* = 0.145 for the risk-ambiguity comparison; $\chi_{1}^{2}=0.045$, *p* = 0.831 for the risk-conflict comparison; and $\chi_{1}^{2}=1.156$, *p* = 0.282 for the ambiguity-conflict comparison). Likewise, accuracy choices were not significantly influenced by sampling format ($\chi_{1}^{2}=0.040$, *p* = 0.842 for the risk-ambiguity comparison; $\chi_{1}^{2}=1.755$, *p* = 0.185 for the risk-conflict comparison; and $\chi_{1}^{2}=2.101$, *p* = 0.147 for the ambiguity-conflict comparison). Thus, there was no discernible effect of type of sampling on degree of ambiguity or conflict aversion.

Hypothesis H2 predicts more pessimistic outcome estimates for the ambiguous and conflicting forecasts than for the risky ones. As in Study 1, participant outcome estimates for each forecast exhibited strong modes. We obtained very similar findings, regardless of whether we counted only responses exactly at the modes, those within 0.25 of the modes, or those within 0.5 of the modes. We present the within 0.25 of the modes version. Midpoint modal responses accounted for 60.6% of the estimates in the risky forecasts. By contrast, only 12.0 and 11.5% of the estimates in the ambiguous and conflictive forecasts were at the midpoint. On the other hand, ambiguous and conflictive forecast estimates were dominated by the lower bounds of the forecasts, with 39.6 and 35.8% of the estimates at the lower bound, respectively, whereas only 6.1% of the estimates in the risky forecasts were at the lower bound. These patterns of results are supportive of hypothesis H2, and an elaboration of appropriate regression models, tests, and findings is available in the Supplementary Materials.

Hypothesis H3, that pessimistic (conservative) prior estimates will increase post-outcome ambiguity and conflict aversion, is tested here by entering the differences between estimates as the predictor of choice of the most accurate forecast (e.g., *d* = risk forecast estimate—ambiguity forecast estimate, so that the model is $y_{ij}^{\prime }=\beta_{0j}+\beta_{1}d_{i}$). This hypothesis is supported for the ambiguity-risk comparison ($\chi_{1}^{2}=4.494$, *p* = 0.034), but not for the conflict-risk condition ($\chi_{1}^{2}=2.895$, *p* = 0.089), nor the conflict-ambiguity comparison ($\chi_{1}^{2}=1.179$, *p* = 0.278).

### Study 2 Discussion

The previous study’s findings that ambiguity and conflict aversion do not disappear when these uncertainties are located in the sample outcomes have been augmented in Study 2 by the evidence that making the sample available sequentially does not seem to have an effect on the strength of aversion. It should be borne in mind that participants knew in advance how many cases would be sampled and they did not have the option of continuing to sample until they decided to stop. Thus, it is possible that sequential sampling with an indefinite number of cases and participants making their own decision to stop might yield effects on aversion (see Güney and Newell, 2015).

In line with the findings from Study 1, Study 2 revealed that ambiguous and conflictive forecasts yielded more pessimistic estimates of the number of days of sunshine than unambiguous forecasts did, but there was no difference between ambiguous and conflictive forecast estimates. However, while prior estimates predicted prior-to-outcome forecaster preferences, they did not predict post-outcome preferences, so the provision of evidence from the outcomes themselves appears to eliminate any impact of participants’ prior estimates regarding the outcomes.

Nevertheless, prior forecast preferences did predict post-outcome choices of the most accurate forecast (and thus post-outcome forecast preferences). The experimental variables’ effects remain largely intact when prior preference is included in the model for predicting the choice of the most accurate forecast, and indeed prior preference is the only predictor that emerges for the conflict-ambiguity condition. Thus prior forecaster preference seems to make an independent contribution to post-outcome choice.

## General Discussion

In two studies, we have shown that ambiguity aversion does not disappear when ambiguity is located in outcomes. We have shown this to be true also of conflict aversion. Moreover, we have found these results hold regardless of whether people are presented with a sample of evidence all at once or if they sample outcomes one at a time, and there seem to be no effects attributable to working memory. Likewise, the degrees of ambiguity and conflict aversion relative to risk appear to be uninfluenced by whether the information is summarized or outcome elaborated and by whether a sample of data is presented sequentially or all at once.

These findings both complement and contrast with those of Dutt et al. (2014), Ert and Trautmann (2014), and Güney and Newell (2015). The studies in the first two articles employ what Güney and Newell (2015, p. 190) call an “outcome sampling” method, whereby unambiguous outcomes are sampled from a fixed but unknown probability distribution. Güney and Newell, on the other hand, use a second-order probability distribution to induce ambiguity, i.e., a “distribution sampling” method. Our method may be referred to as an “ambiguous outcome sampling” (or a “conflictive outcome sampling”) method.

Setting aside the conflictive-uncertainty part of our studies for the moment, why would our findings regarding ambiguity aversion differ from the three previous papers? One explanation is that in all of the studies in those papers, participants could disambiguate the ambiguous alternative by learning the underlying outcome distribution because they were sampling unambiguous outcomes. Although Güney and Newell took steps to address this issue by attempting to prevent the second-order distribution from becoming obvious to participants, they found that substantial percentages of participants nevertheless correctly identified the underlying distributions. Also, as they point out, even participants who could not identify the underlying distribution still could learn what the chances of winning were from the ambiguous alternative, and in particular, they could learn that those chances compared favorably with the risky alternative.

In our setup, samples contained ambiguous outcomes, so when participants took these into account they could not arrive at unambiguous probability estimates. We explored estimates of event probabilities as a candidate explanatory factor for ambiguity and conflict aversion, motivated by the findings in Studies 1 and 2 that such estimates tend to be more “pessimistic” under ambiguity and conflict than under risk. More pessimistic estimates partially accounted for preferences in Study 1. However, they did not predict post-outcome preferences in Study 2, despite the fact that prior forecast preferences did predict post-outcome preferences. Thus, “pessimistic” outcome estimates do not entirely account for ambiguity or conflict aversion when outcomes are indeterminate, so this type of aversion remains to be explained.

Manipulations of amounts of uncertainty in Studies 1 and 2 affected preferences for comparisons between risky and ambiguous or conflictive alternatives, and these effects were not influenced by whether information was summarized or outcome elaborated. So, another candidate explanation is that under ambiguous outcome sampling in our experimental setups, participants can never learn whether the chances of a desirable outcome from the ambiguous alternative are better than, the same as, or worse than the risky alternative. If this inability is a key to ambiguity aversion, then we should find that ambiguity aversion disappears in a setup wherein it is clear to participants that the ambiguous alternative has a higher probability of a desirable outcome than its unambiguous counterpart.

In line with the findings by Hau et al. (2010), the Study 2 results suggest that experiencing the sample all at once versus sequentially does not seem to have an effect on the strength of ambiguity or conflict aversion. As mentioned earlier, the generality of these findings is limited by the fact that participants were not allowed to choose when to stop sampling. It seems plausible that if permitted to sample indefinitely, the presence of ambiguous or conflicting outcomes in samples might motivate people to sample longer than if all outcomes are unambiguous. If that were true, then it is also possible that taking larger samples might decrease ambiguity and conflict aversion (see Güney and Newell, 2015 for preliminary evidence consistent with this speculation). An experiment comparing sampling behavior and preferences under risk, ambiguity, and conflict would appear to be a next logical step in this line of research.

## Ethics Statement

This study was carried out in accordance with the recommendations of The ANU Human Research Ethics Committee with written informed consent from all subjects. All subjects gave written informed consent in accordance with the Declaration of Helsinki. The protocol was approved by The ANU Human Research Ethics Committee, The Australian National University.

## Author Contributions

MS and YS co-designed Study 2. MS conducted the multivariate analyses and took the lead in writing the manuscript and preparing the supplement. DP designed and conducted Study 1 and contributed to the write up. YS conducted Study 2 and contributed to data analysis and preparation of the supplement. BN contributed ideas to the study designs, interpretation of the findings, and the write up.

### Conflict of Interest Statement

The authors declare that the research was conducted in the absence of any commercial or financial relationships that could be construed as a potential conflict of interest.
