# Supplementary material for: Ambiguity and Conflict Aversion When Uncertainty Is in the Outcomes
Source: Front Psychol. 2019 Mar 29;10:539. doi: 10.3389/fpsyg.2019.00539 (PMC6450420; doi:10.3389/fpsyg.2019.00539)
Supplement: Supplementary file 1 [file Data_Sheet_1.PDF]

Ambiguity and Conflict Aversion when Uncertainty is in the Outcomes: Supplementary Material

Michael Smithson, Daniel Priest, Yiyun Shou, and Ben R. Newell

The materials in this document consist of supplementary methods and analyses for Studies 1 and 2, followed by links to code-books for the data-sets for these studies, the data-sets, and documents containing R-code that reproduces all of the statistical analyses pertaining to the results reported in the paper and in the first two parts of this document.

Study 1: Analysis of Best Estimates

As described in the article, we assessed the joint effect of the type of uncertainty (risk, ambiguity, or conflict), and whether the uncertainty is described or experienced, on which of four alternatives participants chose for their "best estimate" of the number of genuine artworks in the bequest via binary logistic regressions that tested three contrasts:

1. midpoint vs lower bound and midway point
2. midway point vs lower bound
3. other value vs everything else

The first two contrasts are designed to compare how conservative or pessimistic participants were about the number of genuine artworks under different experimental conditions, and the third was intended to detect differences in the relative frequency of non-modal choices across experimental conditions. We begin with the midpoint versus lower-bound or midway-point contrast. The mixed logistic regression testing this contrast in the described-versus-experienced and the uncertainty conditions yielded main effects for ambiguity and conflict versus risk and interaction terms for ambiguity and conflict by described-vs-experienced. The fixed-effects part of the model is

$$\begin{aligned} y'_{ij} &= \gamma_0 + \beta_{12}a_i + \beta_{13}c_i + \beta_2 d_i + \beta_{122}a_i d_i + \beta_{132}c_i d_i \\ &= 3.267 - 3.644a_i - 4.166c_i - 0.031d_i - 1.845a_i d_i - 1.229c_i d_i \end{aligned} \tag{S1}$$

where, as before, a and c are binary {0,1} variables identifying the ambiguity and conflict cases, respectively, and d = 1 for the experienced condition and 0 for the descriptive condition. From equation (S1), in the described condition the odds-ratios for choosing the midpoint under ambiguity and conflict versus risk are exp(β_{12}) = exp(-3.644) = 0.026 and exp(β_{13}) = exp(-4.166) = 0.016, respectively, and these ratios are substantially lower in the experienced-uncertainty condition.

The interaction effect arises from the fact that the difference in the popularity of the midpoint between the risk and the other two uncertainty types is much greater in the experienced-information than in the described-information condition. While the described-vs-experienced odds-ratio for risk is close to 1 (from equation (S1), exp(β_2) = exp(-0.031) = 0.969), the corresponding described-vs-experienced odds-ratios for ambiguity and conflict are well below 1 (exp(β_{122}) = exp(-1.845) = 0.158 and exp(β_{132}) = exp(-1.229) = 0.293, respectively).

Turning now to the second contrast, choosing the midway point versus the lower bound for the best estimate, the best mixed logistic regression model produces significant main effects and interaction terms uncertainty type by described-vs-experienced. The fixed-effects part of the model is

$$\begin{aligned} y'_{ij} &= \gamma_0 + \beta_{12}a_i + \beta_{13}c_i + \beta_2 d_i + \beta_{122}a_i d_i + \beta_{132}c_i d_i \\ &= -1.129 - 0.458a_i - 0.683c_i + 0.744d_i - 1.394a_i d_i - 1.202c_i d_i \end{aligned} \tag{S2}$$

The odds_2 column in Table 3 shows that the odds of choosing the midway point rather than the lower bound are lower under ambiguity and conflict than under risk. The effects are not nearly as large as those for the midpoint contrast described above, but they still are substantial. From equation (S2), in the described condition the odds-ratios for choosing the

midpoint under ambiguity and conflict versus risk are $\exp(\beta_{12}) = \exp(-0.458) = 0.633$ and $\exp(\beta_{13}) = \exp(-0.683) = 0.505$, respectively, and these ratios are substantially lower in the experienced-uncertainty condition.

Finally, a mixed logistic regression for the third contrast revealed joint effects of uncertainty type and described-vs-experienced on the odds of choosing a response other than the three modes. The fixed-effects part of the best model is

$$\begin{aligned} y'_{ij} &= \gamma_0 + \beta_{12}a_i + \beta_{13}c_i + \beta_2 d_i + \beta_{122}a_i d_i + \beta_{132}c_i d_i \\ &= -2.340 + 0.932a_i + 1.176c_i + 0.766d_i - 1.027a_i d_i - 0.713c_i d_i \end{aligned} \quad \text{(S3)}$$

As Table 3 in the article shows, a sizeable number of respondents chose values other than the three modes. In the described-uncertainty condition more of them did so under ambiguity and conflict than under risk ($\exp(\beta_{12}) = \exp(0.932) = 2.539$ and $\exp(\beta_{13}) = \exp(1.176) = 3.241$, respectively). However, these differences largely vanished in the experienced-uncertainty condition ($\exp(\beta_{12} + \beta_{122}) = \exp(0.932 – 1.027) = 0.909$ and $\exp(\beta_{13} + \beta_{132}) = \exp(1.176 – 0.713) = 1.588$, respectively).

Study 2: Hypotheses 2 and Minor Hypotheses

We begin with explanations of the stimuli used in Study 2. Figure 2 displays example stimuli for the memory dependent condition. Participants observed two companies’ forecasts as shown in the figure: One for company No.1 and one for company No.2. In this illustration, the participant clicked the button in ③ to purchase a forecast provided by the company No.1, which is shown in cell ②. Each time the participant pressed ③, a new forecast icon would display in ②. The button in ③also displays the number of forecasts remaining to be sampled. The initial number of forecasts is 12, and reduces by 1 each time the participant presses ③. When all forecasts from company No.1 have been purchased, the button in ③ will be disabled and will display the text “all purchased”. Similarly, each time the participant

clicks ⑦, a new forecast provided by the company No. 2 would display in ⑥. The number in ⑦ starts from 12 and reduces by 1 each time the participant presses ⑦ until all forecasts have been purchased.

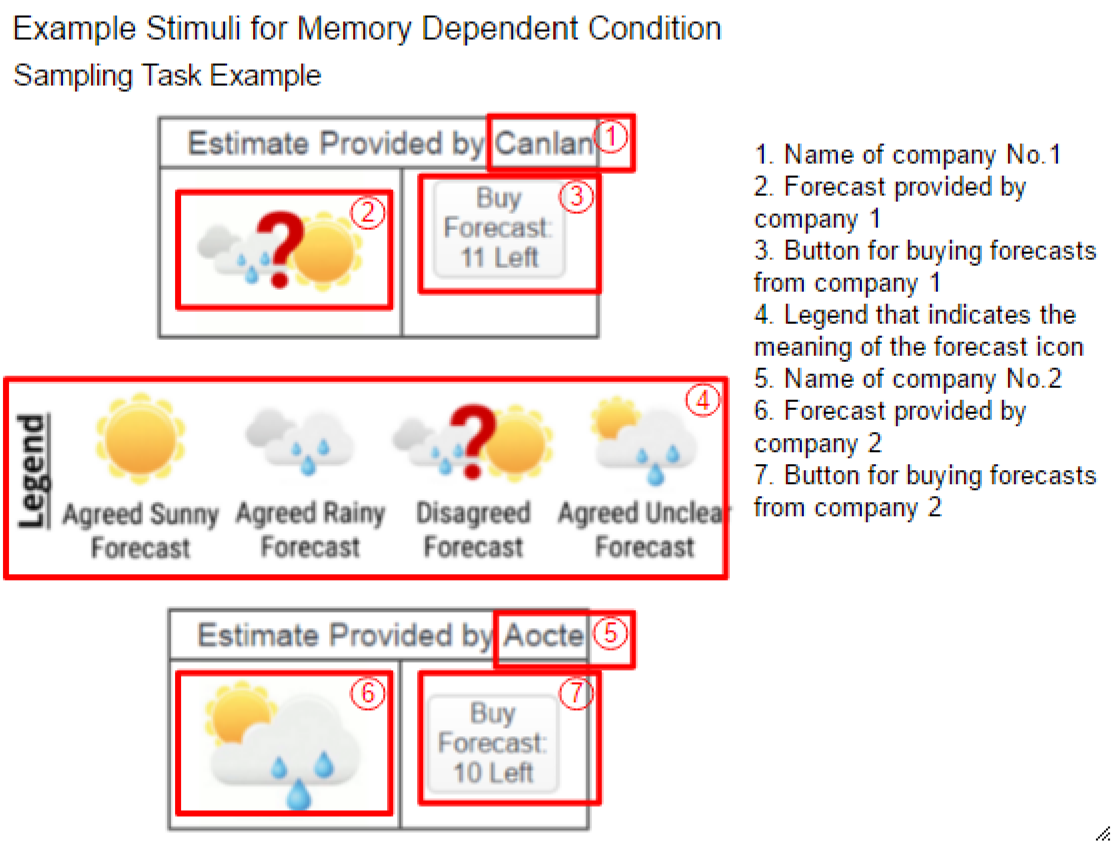

Figure 2. Example stimuli for the memory dependent condition.

Figure 3 displays example stimuli for the memoryless condition. Similar to the memory dependent condition, participants click the button in ③ to purchase a forecast provided by the company No.1 and click ⑥ to purchase a forecast provided by the company No.2. Forecasts from company 1 are displayed in cells in ② according to the type of forecasts, while forecasts from company 2 are displayed in cells in ⑤. The two tables start with empty cells, and add a forecast icon each time participants click the purchase button. The icon stays in the table and additional icons appear when more forecasts are purchased. The figure shows the screen when all forecasts have been purchased and the participant is able to observe all forecasts in one single screen.

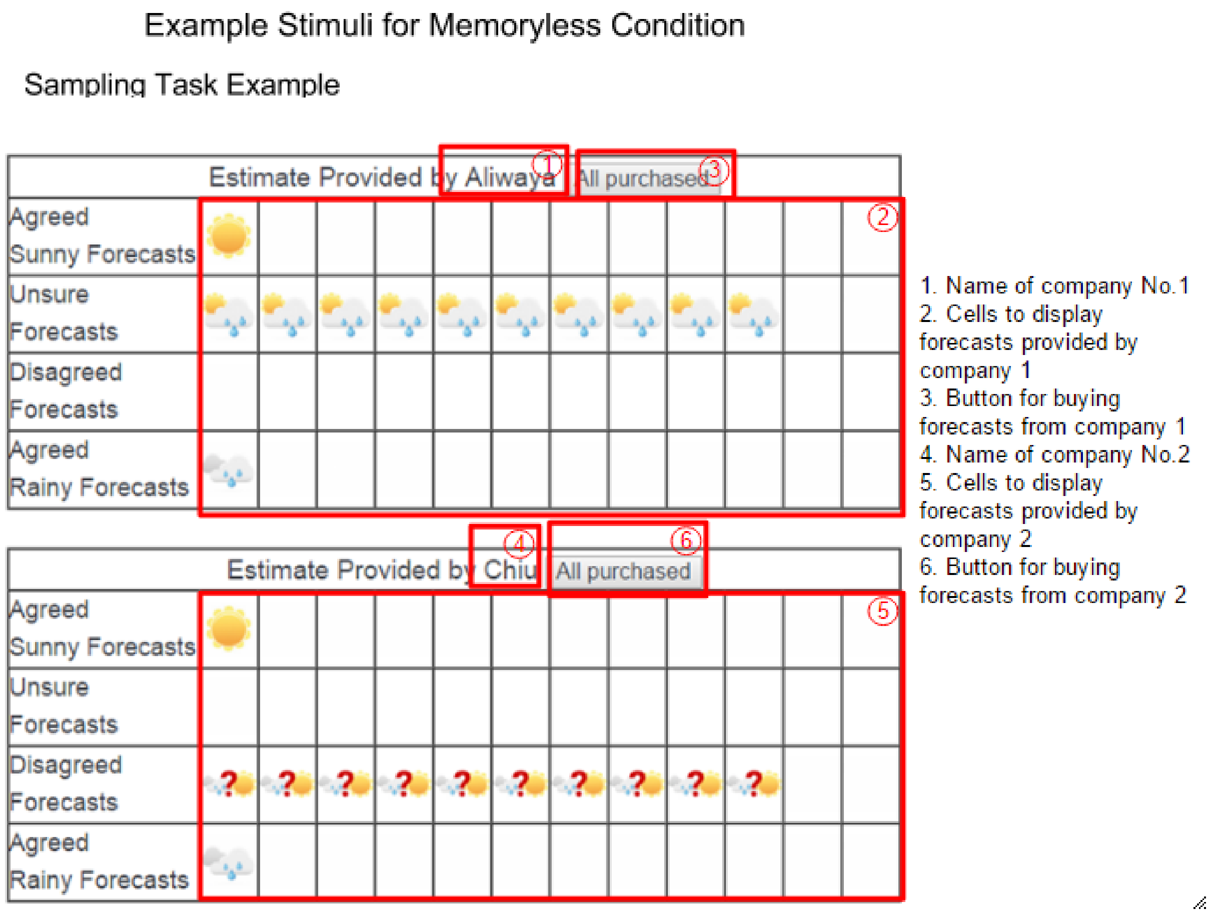

Figure 3. Example stimuli for the memoryless condition

Figure 4 shows sample post-outcome stimuli for Company No.1 in Figure 3. In each cell that displays an icon, the upper left triangle shows the forecast of the company (the same as those in Figure 2), and the lower right triangle with blue background shows the weather outcome.

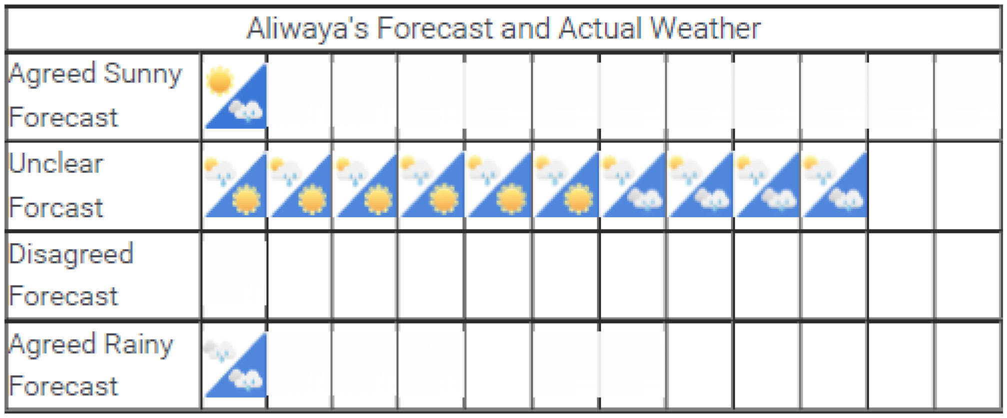

Figure 4: Example post-outcome stimuli.

Hypothesis 2: Ambiguous and conflicting forecasts will result in more pessimistic outcome estimates than risky forecasts

For the overall test of hypothesis H2, given the "lumpy" distribution of the data, we employed ordinal logistic regression with estimates coded as nearest integers from 1 to 7. Combining the risky-ambiguity and risky-conflict conditions, we compared the scores on this integer version of the estimates for the risky forecasts versus the ambiguous and conflictive forecasts combined. When probability of rain, number of uncertain cases, order, and sampling type were included in the model, the conflict-ambiguity effect was moderated by the first three of these predictors whereas sampling type did not produce an effect. The best model is

$$P\left(Y_i \le j\right) = \alpha_j + \beta_1 c_i + \beta_2 r_i + \beta_3 x_i + \beta_4 o_i + \beta_5 c_i r_i + \beta_6 c_i x_i + \beta_7 c_i o_i, \quad \text{(S4)}$$

where $P\left(Y_i \le j\right)$ denotes the cumulative probability that the response falls in or below the jth ordinal category. The α_j are the threshold parameter for the jth cumulative logits (effectively these are "nuisance" parameters). The c_i is a factor variable comparing the risky with the other forecasts, r_i the probability of rain factor with levels "low" and "high", x_i the uncertainty factor with levels "low" and "high", and o_i the order of presentation factor with levels "risky" and "other".

Table S1. Ordinal regression model output

Effect	Coefficient	s.e.	z	p
c = ambig./confl. forecast	-2.187	0.141	-15.552	< .001
r = high prob.	-2.403	0.108	-22.339	< .001
x = low uncert.	-0.098	0.094	-1.044	0.296
o = unambig. first	-0.412	0.198	-2.083	0.037
$c*r$	1.377	0.139	9.905	< .001
$c*x$	-1.103	0.138	-8.005	< .001
$c*o$	0.992	0.136	7.313	< .001

Table S1 displays the relevant output for the ordinal regression model. The effect of *c* (i.e., the cumulative odds-ratio of ambiguous or conflictive to risky estimates) ranges from 4.992 to 82.560, as shown in the odds-ratio (OR) row at the bottom of Table S2. Therefore, hypothesis H2 receives unequivocal support.

Table S2. Relevant odds-ratios from ordinal regression model

	Coeff.	Comp.	P.Rain	Unct.	Order	RainUnct.	RainOrd.	Unct.Ord.	All 3
Company	-2.187	1	1	1	1	1	1	1	1
Prob. Rain	-2.403	0	1	0	0	1	1	0	1
Uncert.	-0.098	0	0	1	0	1	0	1	1
Order	-0.412	0	0	0	1	0	1	1	1
Co.*Rain	1.377	0	1	0	0	1	1	0	1
Co.*Unc.	-1.103	0	0	1	0	1	0	1	1
Co.*Order	0.992	0	0	0	1	0	1	1	1
log odds-ratio	(LOR)	-2.187	-3.212	-3.388	-1.608	-4.414	-2.633	-2.809	-3.834
1/exp(LOR)	(OR)	8.907	24.841	29.603	4.992	82.560	13.921	16.590	46.268

On the other hand, in the ambiguity-conflict comparison, no significant difference was found between estimates for these two types of forecasts. We compared two models:

$$P\left(Y_i \le j\right) = \alpha_j + \beta_2 r_i + \beta_3 x_i + \beta_4 o_i, \tag{S5}$$

and

$$P\left(Y_i \le j\right) = \alpha_j + \beta_1 c_i + \beta_2 r_i + \beta_3 x_i + \beta_4 o_i + \beta_6 c_i r_i + \beta_7 c_i x_i + \beta_8 c_i o_i. \tag{S6}$$

The second model did not significantly improve fit over the first one (the likelihood-ratio test yielded $\chi_4^2 = 7.030$, p = .134).

Ancillary Study 2 Goals

The remaining three goals of Study 2 were as follows. Tests of their hypotheses are presented below.

4. To examine the impact of the amounts of ambiguous or conflictive stimuli on the degree of ambiguity or conflict aversion. We hypothesized that fewer instances of

ambiguity or conflict would decrease the strength of preference for risk over ambiguity or conflict.

5. To examine the impact of the probability of the focal event (e.g., the probability of rain in a weather forecast) on both ambiguity and conflict aversion and on the subjective outcome estimates. We hypothesized that increased probability of the focal event would decrease ambiguity and conflict aversion.
6. To examine perceptions of forecast accuracy as a function of ambiguity, conflict, and the actual outcome. We hypothesized that choosing the most accurate forecast would predict the most preferred forecast after the outcomes are known.

Minor Hypothesis 4: Fewer ambiguous or conflicting cases will decrease ambiguity and conflict aversion

There was only partial support for this hypothesis. It received partial support in both the ambiguity-risk and conflict-risk comparison conditions, with effects in the prior-to-outcome choices (likelihood-ratio tests yield $\chi_1^2 = 12.260$, $p < .001$, and $\chi_1^2 = 10.567$, $p < .001$, respectively) but not in the post-outcome choices ($\chi_1^2 = 0.485$, $p = .486$, and $\chi_1^2 = 3.289$, $p = .070$). Moreover, H4 was not supported in the ambiguity-conflict comparison condition, neither for prior- nor post-outcome choices ($\chi_1^2 = 3.127$, $p = .077$, and $\chi_1^2 = 3.461$, $p = .063$). Table S3 shows the six model likelihood-ratio chi-square tests and the coefficients for the number of ambiguous/conflictive cases. The coefficients' signs are negative in five of the six models, but their magnitudes suggest that if there is an effect it is rather small.

Table S3. H4 Model Chi-Squares and Coefficients

Condition	Prior/Post	χ^2	*p*	Coeff.
Amb.-Risk	Prior	12.260	< .001	-0.670
Amb.-Risk	Post	0.485	.486	-0.130
Conf.-Risk	Prior	10.567	.001	-0.714
Conf.-Risk	Post	3.289	.070	-0.382
Amb.-Conf.	Prior	3.127	.077	0.369

Amb.-Conf.	Post	3.461	.063	-0.415

Minor Hypothesis 5: Greater probability of a focal event (rain) will decrease ambiguity and conflict aversion

Likewise, there was only partial support for this hypothesis. It was unsupported in the ambiguity-risk comparison prior-to-outcome choices but supported in post-outcome choices ($\chi_1^2 = 2.000$, p = .158, and $\chi_1^2 = 5.298$, p = .021). It was supported in both prior- and post-outcome choices in the conflict-risk comparisons ($\chi_1^2 = 9.189$, p = .002, and $\chi_1^2 = 5.984$, p = .014), but not supported in both types of choice in the conflict-ambiguity comparison condition ($\chi_1^2 = 0.270$, p = .604, and $\chi_1^2 = 0.940$, p = .332). Table S4 shows the six model likelihood-ratio chi-square tests and the coefficients for the probability of rain term. The coefficients' signs are negative in four of the six models, but as in H4, their magnitudes suggest that if there is an effect it is small.

Table S4. H5 Model Chi-Squares and Coefficients

Condition	Prior/Post	χ^2	p	Coeff.
Amb.-Risk	Prior	2.000	.158	-0.267
Amb.-Risk	Post	5.298	.021	-0.434
Conf.-Risk	Prior	9.189	.002	-0.664
Conf.-Risk	Post	5.984	.014	-0.517
Amb.-Conf.	Prior	0.270	.604	0.108
Amb.-Conf.	Post	0.940	.332	0.215

Minor Hypothesis 6: Prior forecast preference will predict the choice of which forecast is the more accurate

This hypothesis was tested in models that included effects from the experimental variables. For the combined risk-ambiguity and risk-conflict comparison conditions, the best model turns out to be the model in equation (2) with a main effect for prior-to-outcome choice appended to it. A model including moderator effects did not significantly improve fit ($\chi_4^2 = 8.165$, p = .086). The odds of choosing a forecast as the most accurate was exp(0.795)

= 2.124 times greater if that forecast also had been preferred prior to the weather outcome being known ($p < .001$). Thus, hypothesis H6 was supported.

For the ambiguity-conflict comparison, none of the experimental variables yielded significant effects, so the best model has only an effect from prior forecast choice, and it outperformed the null model ($\chi_1^2 = 9.376$, $p = .002$). The odds of choosing a forecast as the most accurate was exp(0.859) = 2.362 times greater if that forecast also had been preferred prior to the weather outcome being known. Hypothesis H6 was supported for this comparison as well.

Links to Files

Study 1 CodeBook Study 1 Data (long) Study 1 Data (very long)

Study 1 Logistic Regressions Study 1 Table 3

Study 2 CodeBook Study 2 Choice Data Study 2 Estimates Data

Study 2 Analyses
